# Under-reporting of pertussis in Ontario: A Canadian Immunization Research Network (CIRN) study using capture-recapture

**DOI:** 10.1371/journal.pone.0195984

**Published:** 2018-05-02

**Authors:** Natasha S. Crowcroft, Caitlin Johnson, Cynthia Chen, Ye Li, Alex Marchand-Austin, Shelly Bolotin, Kevin Schwartz, Shelley L. Deeks, Frances Jamieson, Steven Drews, Margaret L. Russell, Lawrence W. Svenson, Kimberley Simmonds, Salaheddin M. Mahmud, Jeffrey C. Kwong

**Affiliations:** 1 Public Health Ontario, Toronto, ON, Canada; 2 Department of Laboratory Medicine and Pathobiology, University of Toronto, Toronto, ON, Canada; 3 Dalla Lana School of Public Health, University of Toronto, Toronto, ON, Canada; 4 ProvLab Alberta, Edmonton, AB, Canada; 5 Department of Laboratory Medicine and Pathology, University of Alberta, Edmonton, AB, Canada; 6 Cumming School of Medicine, University of Calgary, Calgary, AB, Canada; 7 Alberta Health, Edmonton, AB, Canada; 8 Division of Preventive Medicine, University of Alberta, Edmonton, AB, Canada; 9 School of Public Health, University of Alberta, Edmonton, AB, Canada; 10 Community Health Sciences, University of Calgary, Calgary, AB, Canada; 11 Vaccine and Drug Evaluation Centre, Max Rady College of Medicine, University of Manitoba, Winnipeg, MB, Canada; 12 Department of Family & Community Medicine, University of Toronto, Toronto, ON, Canada; 13 Institute for Clinical Evaluative Sciences, Toronto, ON, Canada; Universidad Nacional de la Plata, ARGENTINA

## Abstract

**Introduction:**

Under-reporting of pertussis cases is a longstanding challenge. We estimated the true number of pertussis cases in Ontario using multiple data sources, and evaluated the completeness of each source.

**Methods:**

We linked data from multiple sources for the period 2009 to 2015: public health reportable disease surveillance data, public health laboratory data, and health administrative data (hospitalizations, emergency department visits, and physician office visits). To estimate the total number of pertussis cases in Ontario, we used a three-source capture-recapture analysis stratified by age (infants, or aged one year and older) and adjusting for dependency between sources. We used the Bayesian Information Criterion to compare models.

**Results:**

Using probable and confirmed reported cases, laboratory data, and combined hospitalizations/emergency department visits, the estimated total number of cases during the six-year period amongst infants was 924, compared with 545 unique observed cases from all sources. Using the same sources, the estimated total for those aged 1 year and older was 12,883, compared with 3,304 observed cases from all sources. Only 37% of infants and 11% for those aged 1 year and over admitted to hospital or seen in an emergency department for pertussis were reported to public health. Public health reporting sensitivity varied from 2% to 68% depending on age group and the combination of data sources included. Sensitivity of combined hospitalizations and emergency department visits varied from 37% to 49% and of laboratory data from 1% to 50%.

**Conclusions:**

All data sources contribute cases and are complementary, suggesting that the incidence of pertussis is substantially higher than suggested by routine reports. The sensitivity of different data sources varies. Better case identification is required to improve pertussis control in Ontario.

## Introduction

The incidence of pertussis in Canada decreased significantly after the introduction of a whole cell pertussis vaccine in 1943. Since the introduction of acellular pertussis vaccines in 1997, concerns have been raised about lower vaccine effectiveness and shorter duration of immunity leading to outbreaks across Canada, including in the province of Ontario [1,2], albeit none on the scale seen in the United Kingdom (UK) or USA [3,4]. This has led to questions as to whether pertussis is circulating in Ontario at levels higher than detected by any of the multiple existing methods. Understanding the epidemiology of pertussis requires timely, reliable, and accurate surveillance data, but under-diagnosis and under-reporting of pertussis cases are longstanding challenges [5], compromising our ability to accurately estimate the burden of disease. We aimed to estimate the true number of pertussis cases in Ontario separately in infants and those aged one year and over, and evaluate the completeness of each data source through a three-source capture-recapture data analysis.

## Methods

### Data sources

Data sources comprised the integrated Public Health Information System (iPHIS), Public Health Ontario (PHO) Laboratory Information System (Labware), and three healthcare administrative databases held by the Institute for Clinical Evaluative Sciences (ICES): 1) the Canadian Institute for Health Information (CIHI) Discharge Abstracts Database (DAD), which contains information on all hospitalizations in Ontario; 2) the CIHI National Ambulatory Care Reporting System (NACRS), which contains information on all emergency department visits in Ontario; and 3) the Ontario Health Insurance Plan (OHIP) database, which captures physician billing claims, including in primary care (Appendix A in S1_Appendices). For the latter, we only included cases that were billed as being seen in physician offices. Virtually all of Ontario’s 13.9 million residents have publicly funded health insurance through OHIP, with a unique health card number that enables linkage of different healthcare databases. All datasets were linked using a combination of deterministic and probabilistic linkage at ICES to the Registered Persons Database, which contains basic demographic information on all Ontario residents. The unique identifier (health card number) enabled deterministic linkage, and a combination of age, gender, first name, last name and postal code was used for probabilistic linkage if deterministic linkage was not possible. De-identification was done after linkage. Informed consent was not required as data were de-identified; this study was approved by the Public Health Ontario Ethics Review Board #2013–040.01.

iPHIS contains information about all cases of pertussis reported to local public health units in Ontario, where reporting by physicians and laboratories is mandated by the *Health Protection and Promotion Act* 1990. We obtained iPHIS data for pertussis cases meeting either the confirmed or probable case definition, as well as cases that were reported but did not meet either of these case definitions (“Does not meet”) [6] (Appendix B in S1_Appendices). As such, iPHIS contains cases that are laboratory-confirmed as well as those that are not laboratory-confirmed but have been reported because of clinical diagnosis or epidemiological linkage to another confirmed case. We included the “Does not meet” category in order to ensure that we would identify cases recorded as pertussis in other data sources that had not in fact met the provincial case definition, since this would explain administrative cases that did not link to iPHIS and potentially reduce the resulting estimates.

Laboratory data were obtained from PHO, the provincial reference laboratory that conducts >95% of Ontario’s laboratory diagnostic testing for pertussis [2]. In 2009, PHO stopped reporting indeterminate PCR results (defined as threshold cycle values of 36–40) and in 2012, primers targeting a 50bp segment of the recA gene were included to distinguish *B*. *pertussis* from *B*. *holmesii* [2].

The study period was limited to the time range that the PHO laboratory data were available (i.e., from December 7, 2009 to March 31, 2015). Reporting dates to iPHIS may differ from the dates of healthcare encounters and laboratory confirmation. For example, a case may be reported late after a hospitalization has occurred, or laboratory confirmation may be obtained sometime after the case has been reported. In order to avoid missing cases, we also applied a 90-day pre- and post-window for confirming that a case had been captured in a data source. We excluded cases that had an immunization code recorded at the same time as the pertussis billing code (Appendix C in S1_Appendices) because we believed it would be unlikely that a physician would provide an immunization to a patient presenting with a symptomatic pertussis infection.

### Statistical analysis

We used the capture-recapture method, often used for estimating population sizes by analysing the degree of overlap between incomplete sets of cases from different data sources [7,8]. Pertussis cases were ‘captured’ in any database identified from healthcare administrative data and ‘recaptured’ if they appeared in iPHIS or PHO Laboratory Labware (test-positive cases). From iPHIS data we included either confirmed, or confirmed and probable, pertussis cases for all analyses. As a sensitivity analysis we included iPHIS cases that did not meet the case definition (i.e. possible pertussis cases in iPHIS that were not confirmed or probable). We took three approaches to the capture-recapture analysis with respect to administrative data, using Labware and iPHIS data in all three, but adding DAD, DAD/NACRS, or DAD/NACRS/OHIP.

To estimate the number of cases that were not found by any of the sources of data, we used a three-source capture recapture hierarchical model in order to enable lower order interactions and main effects of the interaction to be examined, which accounts for dependencies. This method adjusted for dependency among sources by including their interaction terms in the Poisson model assuming a closed population. Models with all possible two-way interactions were fitted and the optimal models were selected based on the lowest Bayesian Information Criterion (BIC) value [9]. Abundance was estimated with maximum likelihood with the Poisson model. Data linkages were performed in SAS version 9.3 (The SAS Institute, Cary, NC) and estimations were done in R (R capture package) [10]. The sensitivity of each source was calculated as the number of cases identified by a source as a percentage of the estimated total number of cases (i.e., combining those from all sources plus those not identified by any of the sources). We looked for dependency between sources by calculating the probability of a case being identified in one data source given that it appears in another source, and testing the hypothesis that the cases are randomly detected in each source by chi-squared test of two by two tables of the observed number of cases in a pair of sources compared with the estimated totals using an alpha of 5%.

We stratified the analysis by two age groups (infants [i.e., younger than one year of age] and individuals aged one year and over) because the severity of disease and likelihood of interaction with healthcare, particularly hospitalizations and emergency department visits, is much higher among infants [1]. In a sensitivity analysis to understand the impact of potential over-ascertainment of cases by administrative data (cases mistakenly labelled or miscoded as pertussis) we calculated the expected number of true positive cases assuming that the true positive cases were 25%, 50%, or 75% of cases identified by administrative data. We used confirmed and probable cases in iPHIS for the sensitivity analysis to ensure maximum sensitivity of the public health data. As a comparison, we also include this sensitivity analysis using confirmed iPHIS cases in the supplementary material (Appendix D in S1_Appendices).

## Results

**Fig 1:**
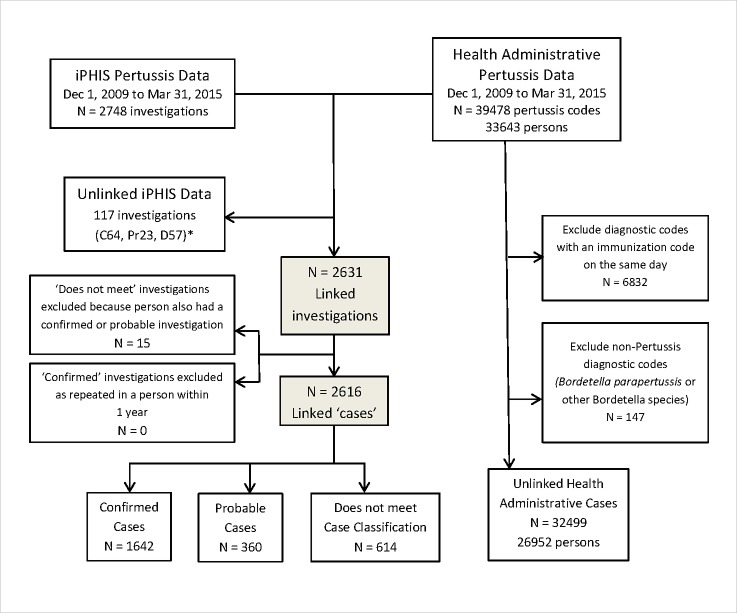
Data flow chart. *C = confirmed, P = probable, D = “Does not meet” case definition.

**Fig 2:**
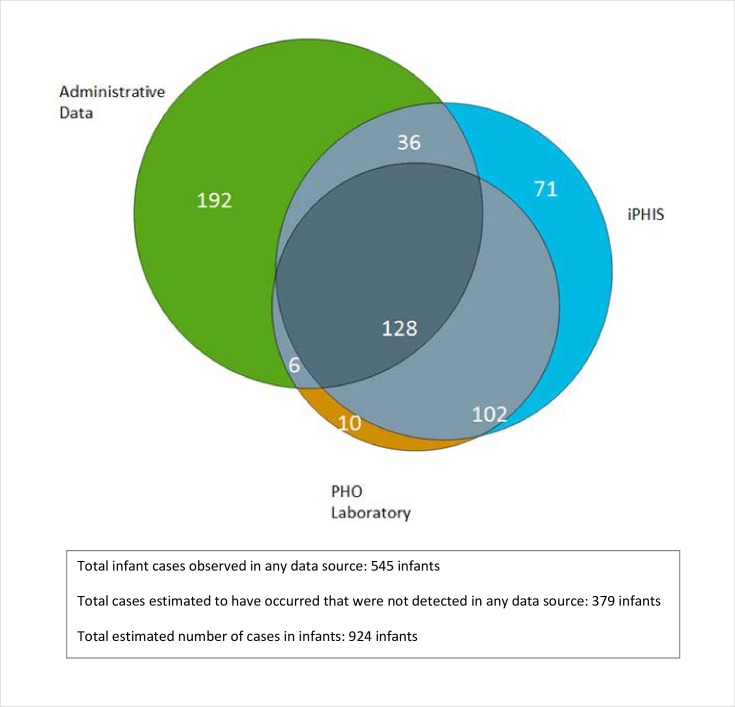
Overlap of data sources. Example for infants, confirmed and probable cases in iPHIS, compared with laboratory-confirmed cases and healthcare administrative data (DAD and NACRS).

Overall, 95.7% of iPHIS cases were linked successfully with administrative data (Fig 1). The distribution of the total number of pertussis cases from all three data sources and their dependencies are schematically illustrated for the example of confirmed and probable cases among infants identified through iPHIS, DAD/NACRS, and Laboratory data (Fig 2). We observed a total of 545 infant cases, including 128 found in all sources, shown in the central area of overlap. An additional 379 cases were estimated to be undetected by any source (lying outside all the circles), bringing the total estimate from the capture-recapture analysis of this combination of data to 924 cases. The total numbers in each source for this example were 337 iPHiS cases, 246 laboratory cases, and 362 administrative data cases (Table 1).

**Table 1:** Capture-recapture analysis results for infants in Ontario from Dec 7, 2009 to Mar 31, 2015.

Data source	Cases included	Total Observed	Estimated Total Number	Confidence Limits	Model interactions
**DAD vs. iPHIS vs. PHO Laboratory data**	Confirmed cases only	373	431	(407, 460)	iPHIS and Laboratory data
	Confirmed and probable	392	497	(448, 569)	Administrative and Laboratory data; iPHIS and Laboratory data
**DAD/NACRS vs. iPHIS vs. PHO Laboratory Data**	Confirmed cases only	528	859	(730, 1051)	Administrative and Laboratory data; iPHIS and Laboratory data
	Confirmed and probable	545	924	(786, 1126)	Administrative and Laboratory data; iPHIS and Laboratory data
**DAD/NACRS/OHIP vs. iPHIS vs. PHO Laboratory data**	Confirmed cases only	1528	2752	(2308, 3392)	Administrative and Laboratory data; iPHIS and Laboratory data
	Confirmed and probable	1541	2872	(2431, 3488)	Administrative and Laboratory data; iPHIS and Laboratory data
	Confirmed, probable, and “does not meet”	1583	3081	(2675, 3614)	Administrative and Laboratory data; iPHIS and Laboratory data

For the model using Laboratory, iPHIS confirmed and probable cases and DAD/NACRS administrative data, laboratory data captured only 1% more cases beyond administrative and iPHIS data (10/924), while administrative data captured 21% (192/924) more cases and iPHIS data 7.7% (71/924). Similar results were found for analyses including DAD data only or DAD/NACRS/OHIP data and for the >1 year age group.

Dependency between iPHIS and laboratory data is shown visually for the model including infants and DAD/NACRS administrative data by the degree of overlap of the circles (Fig 2). The dependency between data sources is illustrated for confirmed and probable cases in iPHIS using two different administrative data models, with or without OHIP data, and the two age groupings in Appendix E in S1_Appendices. The proportion of cases identified by the laboratory data that were not found as confirmed or probable cases in iPHIS was relatively low and varied only slightly from 2.5% (in those more than a year old, using DAD/NACRS and OHIP) to 6.5% (in those less than one year of age using DAD/NACRS) (Appendix E in S1_Appendices). The proportion of laboratory-identified cases not identified in administrative data varied more widely from 27.5% in those less than one year of age using DAD/NACRS/OHIP to 84% in those over one year of age using DAD/NACRS (Appendix E in S1 Appendices).

Because a strong association was found between PHO Laboratory and iPHIS data (e.g. Chi-squared 470.5; p<0.00001 for infants) we therefore adjusted for this dependency in the modelling. The best-fitting models consistently included interactions between laboratory and both administrative and iPHIS data sources (Tables 1 and 2). Within each age group stratum, total estimates increased stepwise with (i) the inclusion of probable in addition to confirmed iPHIS cases and (ii) the addition of each extra administrative data source from DAD, DAD/NACRS, through to DAD/NACRS/OHIP (Tables 1 and 2). Adding probable cases increased each estimate by a small amount; adding NACRS data made a greater difference, increasing total estimates by about two-fold for infants and four-fold for those aged 1 year and over. Adding the “Does not meet” category increased the total estimated number of cases in all models, rather than reducing it, and did not therefore explain the unlinked administrative cases. The greatest step up in the estimated number of cases was given by adding OHIP data to the model.

**Table 2:** Capture-recapture results for those aged one year and over in Ontario from Dec 7, 2009 to Mar 31, 2015.

Data source	Cases included	Total Observed	Estimated Total Number	Confidence Limits	Model interactions
**DAD vs. iPHIS vs. PHO Laboratory data**	Confirmed cases only	1399	3604	(2479, 5831)	iPHIS and Laboratory data
	Confirmed and probable	1735	4483	(3083, 7256)	iPHIS and Laboratory data
**DAD/NACRS vs. iPHIS vs. PHO Laboratory Data**	Confirmed cases only	3005	12105	(10796, 13659)	iPHIS and Laboratory data
	Confirmed and probable	3304	12883	(11593, 14395)	iPHIS and Laboratory data
**DAD/NACRS/OHIP vs. iPHIS vs. PHO Laboratory data**	Confirmed cases only	26484	92128	(79178, 108548)	Administrative and Laboratory data; iPHIS and Laboratory data
	Confirmed and probable	26680	68891	(64898, 73288)	iPHIS and Laboratory data
	Confirmed, probable, and “does not meet”	27059	83921	(77423, 91294)	Administrative and Laboratory data; iPHIS and Laboratory data

The sensitivity of individual data sources varied markedly by data source and age group (Table 3). Sensitivity was highest at 68% for confirmed and probable cases among infants reported to iPHIS. However, iPHIS was not sensitive for the older age group, dropping to a level of 2% using estimates based on hospitalization, emergency department and physician office visit data. Hospitalizations were most sensitive for infants (40%), and not at all sensitive for the older age group (1%). Adding emergency department data did not dramatically impact sensitivity for infants (47% versus 40%) but did increase sensitivity for the group aged 1 and older, increasing from 1% to 15%. Laboratory data reached their highest sensitivity of 50% for hospitalized (DAD) infants, but when emergency department (NACRS) data were included, sensitivity dropped to 27% and with OHIP data included, sensitivity fell to 9%.

**Table 3:** Sensitivity of different data sources in Ontario from Dec 7, 2009 to Mar 31, 2015 based on DAD or DAD/NACRS or DAD/NACRS/OHIP, iPHIS confirmed and probable cases and PHO Laboratory data.

	Data source	Sensitivity (%, n/N)	95% Confidence interval (%)
Infants, DAD only	iPHIS	68% (337/497)	64 to 72
	DAD	40% (200/497)	35 to 44
	Laboratory	50% (247/497)	27 to 55
	All sources combined (DAD/iPHIS/Laboratory)	79% (392/497)	75 to 81
Infants, DAD/NACRS	iPHIS	36% (337/924)	33 to 39
	DAD/NACRS	47% (434/924)	43 to 50
	Laboratory	27% (247/924)	24 to 30
	All sources combined (DAD/NACRS/iPHIS/Laboratory)	59% (545/924)	55 to 62
Infants, DAD/NACRS/OHIP	iPHIS	12% (337/2872)	11 to 13
	DAD/NACRS/OHIP	49% (1417/2872)	47 to 51
	Laboratory	9% (247/2872)	8 to 10
	All sources combined (DAD/NACRS/OHIP/iPHIS/Laboratory)	54% (1541/2872)	52 to 56
Aged 1 year and over DAD only	iPHIS	37% (1665/4483)	36 to 39
	DAD	1% (36/4483)	0.6 to 1.3
	Laboratory	22% (987/4483)	21 to 24
	All sources combined (DAD/iPHIS/Laboratory)	39% (1735/4483)	38 to 41
Aged 1 year and over, DAD/NACRS	iPHIS	13% (1665/12883)	12 to 14
	DAD/NACRS	15% (1885/12883)	14 to 15
	Laboratory	8% (987/12883)	7 to 9
	All sources combined (DAD/NACRS/iPHIS/Laboratory)	27% (3304/12883)	26 to 28
Aged 1 year and over, DAD/NACRS/OHIP	iPHIS	2% (1665/68891)	2 to 2.5
	DAD/NACRS/OHIP	37% (25607/68891)	36 to 38
	Laboratory	1% (987/68891)	0.7 to 1.1
	All sources combined (DAD/NACRS/OHIP/iPHIS/Laboratory)	39% (26680/68891)	38 to 40

If hospitalization and emergency department data are over-ascertaining cases such that only 25% of unlinked cases were valid, estimated cases in infants would be a little over halved (924 cases down to 496 cases) (Table 4). In those aged 1 year and over, the corresponding proportionate decrease would be a reduction down to 35% of the original estimate (12883 cases down to 4510 cases). The impact in a similar analysis using OHIP data showed a greater proportional impact because the number of cases in OHIP was higher than in other sources. Among those aged <1 year, the reduction in the numbers would be down to a little over a third (2872 cases down to 983 cases) and in the older age groups it would be down to 27% of the original estimates (68891 cases down to 18510 cases). However, even if the proportion of valid cases was only 25%, the absolute numbers remain very high, with more than 5000 additional cases than would be estimated from hospitalization and emergency department data assuming those sources were 100% true cases.

**Table 4:** Estimates of total cases in Ontario from Dec 7, 2009 to Mar 31, 2015, adjusted for different proportions of true positive cases in unlinked administrative data, and using confirmed and probable cases in iPHIS.

Age Group	Data Source	Proportion of unlinked administrative cases that are true positives			
		25%	50%	75%	100%
Infants	DAD+NACRS	496	638	781	924
	DAD+NACRS+OHIP	983	1612	2242	2872
Aged 1 year and older	DAD+NACRS	4510	7299	10094	12883
	DAD+NACRS+OHIP	18510	35302	52098	68891

## Discussion

Our findings indicate that none of the sources of information on pertussis in Ontario is complete. We found the best sensitivity was 68% for cases in infants reported to public health. All other sources, including any source for the older age group had worse sensitivity. Our findings are validated by similar findings recently published for the Netherlands where sensitivity of different databases varies and is highest in younger age groups [8,11]. Pertussis is recognized in many jurisdictions to be under-diagnosed, under-investigated, and under-reported [12,13]. We have no reason to suspect that Ontario is different in this regard. This is probably due to a variety of factors. It can be difficult for physicians to distinguish mild pertussis from other acute respiratory infections, or they may choose not to test, or they may record symptoms rather than a suspected diagnosis, or they may not report a suspected diagnosis to the public health department. A laboratory result may not be reported, or the clinical symptoms that are required for the provincial case definition may not be present in the patient, particularly for older children and adults. We did not adjust our findings in light of the 5% of laboratory testing that were estimated not to be undertaken by PHO; however we think that this would not have any meaningful impact on the results because the proportion is so low.

Although Ontario’s probable case definition is more sensitive than the confirmed case definition, only 17% of all reports were probable cases, which may indicate that physicians are under-investigating and under-reporting given that we would not expect a high proportion of cases in older patients with prolonged cough to be laboratory-confirmed by PCR only [14]. Efforts to increase physician reporting of probable cases may be warranted.

Our results should also lead to consideration of whether the Ontario confirmed and probable case definitions are calibrated too much in favour of high specificity at the expense of sensitivity. In infants this may not be such an issue because they are more likely to have typical symptoms, to be hospitalized and to be tested. Hence iPHIS was highly sensitive for infants. However, sensitivity of reporting to public health became extremely poor in the older age group. Pertussis can be quite mild in older individuals and present with non-specific cough without paroxysm, vomiting, or apnoea. In the US and Ontario, clinical case definitions seem to favour specificity over sensitivity because they require at least one typical symptom of pertussis (cough lasting 2 weeks or longer, paroxysmal cough, cough with inspiratory "whoop", or cough ending in vomiting or gagging, or associated with apnea) [15]. Case definitions recommended for surveillance by the World Health Organization [16], the European Centre for Disease Control and the UK [17] include physician-diagnosed pertussis and therefore may have greater sensitivity. The UK public health laboratory also provides serology and oral fluid as more sensitive diagnostic methods for older patients [18]. Case definitions that are more specific than sensitive may delay early reporting of outbreaks, lead to under-estimates of level of disease, a false sense of security about how well pertussis is controlled, and over-estimates of vaccine effectiveness [19].

Some support for our results is given by a recent mathematical modelling analysis suggesting that the incidence of pertussis in Ontario may be much higher than is reflected in numbers meeting the reportable disease case definitions [20]. The model suggested extremely high levels of under-reporting for those aged 1 year and over, increasing from around 1 case reported for every 600 undetected pertussis cases in 2- to 7-year olds up to approximately 1 in 33,000 cases in the 20- to 64-year old age group. Our estimates are relatively modest compared with these independent estimates derived through mathematical modelling. The largest estimates came from adding OHIP data for both age groups. Compared with DAD/NACRS, OHIP data increased estimates for those aged 1 year and over between 5- and 8-fold, up to the highest estimate of nearly 69,000 cases, which equates to around 13,000 per year. While we are inclined to question the plausibility of using OHIP data, results are within the range given by this modelling approach. The higher estimates are also in line with numbers observed in Australia, where average annual numbers have exceeded 22,000 cases [21] in a population 1.7 times the population of Ontario (which would equate to 13,000 cases per year in Ontario), but higher than seen in England where cases have peaked at less than 10,000 cases per year [22] in a population nearly four times the size of Ontario (which would equate to around 2,500 cases per year in Ontario). We wondered whether the OHIP cases might be true cases with fewer symptoms that would not meet confirmed or probable case definitions. However, adding the iPHIS cases that did not meet the case definition to the analysis did not substantially reduce the estimates (Table 2). Interestingly, widespread outbreaks have not been reported in Ontario, unlike in many other jurisdictions that have been using acellular pertussis vaccines for as long as Ontario.

Capture-recapture analysis is one approach to examining potential under-reporting in order to improve understanding of the epidemiology of pertussis. Capture-recapture analysis relies on a number of assumptions which are rarely met in applying this method to health data, but which are not absolute. Each case should be diagnosed accurately, matching of cases done appropriately, and the cases that are included are within the time-space unit under study. For any single source, each case in the population needs to have the same ‘catchability’ or probability of ascertainment, and ascertainment of any case by the sources should be independent [23–25]. Finally, our methods assumed that the population under study was closed without large-scale movements in or out. Data sources are rarely perfect, particularly with respect to independence of health and surveillance data, which is the most common application of this method (7, 9). As also illustrated visually in Fig 2, dependency was greatest between laboratory and iPHIS data and less marked between the other data sources. The extent of overlap is similar in younger and older age groups as well as when OHIP data are added to the model (Appendix E in S1_Appendices). For those familiar with laboratory and public health surveillance data, the dependence we observed is unsurprising since laboratory confirmation frequently triggers reporting, and this is also legally mandated in many jurisdictions including Ontario. Without adjustment, this dependence risks under-estimating the “true” number of cases of pertussis. By using a three-source approach, we were able to adjust for lack of independence while maintaining the value of keeping them separate, as this contributes to the estimate of overall abundance. We used a sensitivity analysis to examine the effect of potential issues with miscoding of administrative data. To address different catchability we stratified by age because although the diagnosis can be missed in infants, they are more likely than older age groups to be symptomatic, tested, and hospitalized. Catchability could also vary by other factors not considered in our analysis such as socioeconomic status or access to healthcare. Multiple sources are independent if the probability of a member of the population being in the overlap of any particular subset of all the sources is equal to the product of the overall probabilities of being in any one source within this subset. We found dependency between data sources, which was expected–for example, reporting by laboratories of confirmed cases is not only a legislative requirement but also often triggers reporting to public health by physicians as well as, potentially, billing codes in OHIP. This dependency was addressed by log-linear modelling [9].

The greatest limitation of this study rests with the lack of validated healthcare administrative data. This seemed to be a particular issue with OHIP data. For example, considering the estimate of 2,872 cases based on analysis of hospitalization, emergency department, and physician billing data, we would have expected approximately 5.7 deaths to occur during the study period of 5.25 years, or around 1 death per year, using an estimated case fatality rate of ~0.2% [26]. Yet there have been no confirmed deaths from pertussis in Ontario reported during the study period. Under-reporting of pertussis deaths may occur, perhaps because they are misclassified as sudden infant deaths or an undiagnosed cause, but they are more likely to be reported than milder cases [11, 27]. We believe that the large numbers estimated from OHIP data are therefore unlikely to be valid, or at least do not seem to be important with respect to the primary goal of the immunization program which is to protect infants from severe disease. False-positive cases may have arisen from misdiagnosis or coding error, for example, because pertussis was recorded by mistake or as part of a differential diagnosis that was not confirmed, or used mistakenly instead of the code for pertussis vaccination administration or counselling. Such miscoding may be less likely for emergency department visits and even less so for hospitalization, reflected in what is likely to be an increasing specificity for each source. These explanations are speculative and highlight the need for a validation study of the administrative data to examine what is in the complete clinical record of the cases [28]. The underlying purpose of OHIP data is physician reimbursement, not disease surveillance, and while the use of OHIP and other un-validated administrative data have potential to be useful in identifying exposure and disease outcomes, significant limitations of such data have been described [29–31] and indicate that caution is advised. OHIP data are likely to include both false positive pertussis cases, which lead to over-estimation in capture-recapture analysis, and false negative cases, which are less of an issue with this method unless they are systematically different in some way from cases identified in other sources.

The burden of pertussis in Ontario is likely to be much higher than routine data indicate. Further study is needed on how best to optimize methods of estimating the incidence of pertussis in Ontario. In the context of ongoing challenges in controlling pertussis, improvements in the quality of surveillance are needed in order to strengthen the evidence base for decision making about pertussis immunization in Ontario.

## Supporting information

S1 Appendices **Appendix A:** Data Source Abbreviations and Descriptions.
**Appendix B**: Data Case definitions for data sources.
**Appendix C:** Immunization codes (used to exclude cases).
**Appendix D:** Sensitivity of different data sources in Ontario from Dec 7, 2009 to Mar 31, 2015 based on DAD or DAD/NACRS or DAD/NACRS/OHIP, iPHIS confirmed cases and PHO Laboratory data.
**Appendix E:** Proportion of laboratory confirmed cases not found in other data sources, by age and combination of administrative data sources.
(DOC)
