# Supplementary material for: Under-reporting of pertussis in Ontario: A Canadian Immunization Research Network (CIRN) study using capture-recapture
Source: PLoS One. 2018 May 2;13(5):e0195984. doi: 10.1371/journal.pone.0195984 (PMC5931792; doi:10.1371/journal.pone.0195984)
Supplement: S1 Appendices — Appendix A: Data Source Abbreviations and Descriptions. Appendix B: Data Case definitions for data sources. Appendix C: Immunization codes (used to exclude cases). Appendix D: Sensitivity of different data sources in Ontario from Dec 7, 2009 to Mar 31, 2015 based on DAD or DAD/NACRS or DAD/NACRS/OHIP, iPHIS confirmed cases and PHO Laboratory data. Appendix E: Proportion of laboratory confirmed cases not found in other data sources, by age and combination of administrative data sources. (DOC) [file pone.0195984.s001.doc]

**Appendix A**: Data Source Abbreviations and Descriptions

| **Data Source Name** | **Abbreviation** | **Description** |
| --- | --- | --- |
| Integrated Public Health Information System | iPHIS | Ontario’s reportable disease database, containing information about investigations of diseases by physicians and laboratories as mandated by the *Health Protection and Promotion Act,* 1990. |
| Public Health Ontario Laboratory Information System | PHO Labware | Ontario’s provincial reference laboratory system that contains information on >95% of the diagnostic testing for pertussis in the province |
| Registered Persons Database | RPD | A population based registry of unique health numbers for persons eligible for coverage under the Ontario Health Insurance Plan |
| Canadian Institute for Health Information Discharge Abstracts Database | CIHI-DAD | The DAD contains demographic, administrative and clinical data for hospital inpatient discharges and day surgeries |
| Canadian Institute for Health Information National Ambulatory Care Reporting System | CIHI-NACRS | NACRS contains demographic, administrative and clinical data for emergency department (ED) visits |
| Ontario Health Insurance Plan database | OHIP | Database containing all claims made by physicians and other health care providers for insured services provided to Ontario residents |

**Appendix B:** Data Case definitions for data sources

| **Variable** | **Definition** |
| --- | --- |
| **PHO Laboratory Case Definition**  *Data Source:* PHO Labware | **Positive Case**: Cases with at least one test result positive by PCR or culture  *Test = PCR. Tests>90 days apart considered to be a new record* |
| **Reportable Disease Case Definition**  *Data Source:* iPHIS | **Confirmed Case:** Laboratory confirmation of infection: Isolation of *Bordetella pertussis* from an appropriate clinical specimen (e.g., nasopharyngeal swabs) OR Detection of *B. pertussis* deoxyribonucleic acid (DNA) by nucleic acid amplification test (NAAT)) from an appropriate clinical specimen (e.g., nasopharyngeal swabs) AND one or more of the following:  • cough lasting 2 weeks or longer  • paroxysmal cough of any duration  • cough with inspiratory "whoop"  • cough ending in vomiting or gagging, or associated with apnea  OR Epidemiologic link to a laboratory-confirmed case AND one or more of the following for which there is no other known cause:  • paroxysmal cough of any duration  • cough with inspiratory "whoop"  • cough ending in vomiting or gagging, or associated with apnea  **Probable Case:** Cough lasting 2 weeks or longer in the absence of appropriate laboratory tests and not epidemiologically linked to a laboratory-confirmed case for which there is no other known cause AND one or more of the following, with no other known cause:  • paroxysmal cough of any duration  • cough with inspiratory "whoop"  • cough ending in vomiting or gagging, or associated with apnea |
| **Administrative Data Case Definition**  *Data Sources:*  CIHI-DAD  CIHI-NACRS  OHIP | **Positive Case**: Cases with at least one of the following codes will be considered a pertussis case:   - OHIP code (based on ICD-9) 033 Pertussis - DAD/NACRS ICD-10th Revision A37.0 Whooping cough due to *Bordetella pertussis*   Excluded:   - non-pertussis diagnostic codes ICD-10 A37.1 (*Bordetella parapertussis*) and A37.8 (Other Bordetella species) (n=147) see Fig 1. - diagnostic code associated with an immunization code on the same day (n=6,832) see Fig 1. |

**Appendix C**: Immunization codes (used to exclude cases)

| **Info.** | **Code** | **Sub Category** | **Description** |
| --- | --- | --- | --- |
| Introduced 2011  Procedural, specific | **G840** | Active Immunizing Agents | Diphtheria-Tetanus-acellular Pertussis-Inactivated Polio; DTaP IPV |
|  | **G841** |  | Diphtheria-Tetanus-acellular Pertussis-Inactivated Polio-*Haemophilus influenza*  type B; DTaP-IPV-Hib |
|  | **G847** |  | Tetanus-diphtheria-acellular pertussis; Tdap |
| Revised 2011  Generic, procedural | **G538** | Active Immunization-Injection of unspecified agent | With visit (each injection) |
| Deleted 2011  Generic, procedural | **G539** | Active Immunization-Injection of unspecified agent | Sole reason (first injection) |

**Appendix D:** Sensitivity of different data sources in Ontario from Dec 7, 2009 to Mar 31, 2015,

**based on DAD or DAD/NACRS or DAD/NACRS/OHIP, iPHIS confirmed cases and PHO Laboratory data.**

|  | **Data source** | **Sensitivity (%, n/N)** | **95% Confidence interval (%)** |
| --- | --- | --- | --- |
| Infants, DAD only | iPHIS | 73% (316/431) | 69 to 77 |
|  | DAD | 47% (200/431) | 42 to 51 |
|  | Laboratory | 57% (247/431) | 32 to 62 |
|  | All sources combined (DAD/iPHIS/Laboratory) | 87% (373/431) | 83 to 89 |
| Infants, DAD/NACRS | iPHIS | 37% (316/859) | 34 to 40 |
|  | DAD/NACRS | 51% (434/859) | 47 to 54 |
|  | Laboratory | 29% (247/859) | 26 to 32 |
|  | All sources combined (DAD/NACRS/iPHIS/Laboratory) | 62% (528 / 859) | 58 to 65 |
| Infants, DAD/NACRS/OHIP | iPHIS | 11% (316/2752) | 10 to 12 |
|  | DAD/NACRS/OHIP | 51% (1417/2752) | 49 to 53 |
|  | Laboratory | 9% (247/2752) | 8 to 10 |
|  | All sources combined (DAD/NACRS/OHIP/iPHIS/Laboratory) | 56% (1528/2752) | 54 to 58 |
| Aged 1 year and over DAD only | iPHIS | 37% (1326/3604) | 35 to 38 |
|  | DAD | 1% (36/3604) | 0.7 to 1.4 |
|  | Laboratory | 27% (987/3604) | 26 to 29 |
|  | All sources combined (DAD/iPHIS/Laboratory) | 39% (1399/3604) | 37 to 40 |
| Aged 1 year and over, DAD/NACRS | iPHIS | 11% (1326/12105) | 10 to 12 |
|  | DAD/NACRS | 16% (1885/12105) | 15 to 16 |
|  | Laboratory | 8% (987/12105) | 8 to 9 |
|  | All sources combined (DAD/NACRS/iPHIS/Laboratory) | 25% (3005 / 12105) | 24 to 26 |
| Aged 1 year and over, DAD/NACRS/OHIP | iPHIS | 1% (1326/92128) | 1 to 1.5 |
|  | DAD/NACRS/OHIP | 28% (25607/92128) | 27 to 29 |
|  | Laboratory | 1% (987/92128) | 0.8 to 1.1 |
|  | All sources combined (DAD/NACRS/OHIP/iPHIS/Laboratory) | 29% (26484/92128) | 28 to 30 |

**Appendix E:** Proportion of laboratory confirmed cases not found in other data sources, by age and combination of administrative data sources

| **Age group** | **less than 1 year of age** | | **more than 1 year of age** | |
| --- | --- | --- | --- | --- |
| **Administrative data sources: DAD & NACRS data** | without OHIP | with OHIP | without OHIP | with OHIP |
|  | % | % | % | % |
| **Proportion laboratory confirmed cases not found in iPHIS** | 6.5% | 4.5% | 4.9% | 2.5% |
| **Proportion laboratory confirmed cases not found in administrative data** | 45.5% | 27.5% | 84.0% | 60.0% |
